# Safe and effective person- and family-centered care practices during transitions from hospital to home—A web-based Delphi technique

**DOI:** 10.1371/journal.pone.0211024

**Published:** 2019-01-22

**Authors:** Chantal Backman, Sharon Johnston, Nelly D. Oelke, Katharina Kovacs Burns, Linda Hughes, Wendy Gifford, Jeanie Lacroix, Alan J. Forster

**Affiliations:** 1 School of Nursing, Faculty of Health Sciences, University of Ottawa, Ottawa, Canada; 2 Clinical Epidemiology Program, Ottawa Hospital Research Institute, Ottawa, Canada; 3 Bruyère Research Institute, Ottawa, Canada; 4 Faculty of Medicine, University of Ottawa, Ottawa, Canada; 5 School of Nursing, Faculty of Health and Social Development, University of British Columbia, Kelowna, Canada; 6 Patients for Patient Safety Canada, Edmonton, Canada; 7 School of Public Health, University of Alberta, Edmonton, Canada; 8 Canadian Institute for Health Information, Toronto, Canada; University of Auckland, NEW ZEALAND

## Abstract

**Background:**

Research has shown that adverse events during care transitions from hospital to home can have a significant impact on patients’ outcomes, leading to readmission, delayed healing or even death. Gaps exist in the ways of monitoring care during transition periods and there is a need to help organizations better implement and monitor safe person-and family-centered care. Value statements are a way to obtain narratives in lay terms about how well care, treatment and support is organized to meet the needs and preferences of patients/families. The purpose of this study was to identify the value statements that are perceived by decision-makers and patients/families to best signify safe person- and family-centered care during transitions from hospital to home.

**Methods:**

Between January and September 2017, a web-based Delphi was used to survey key stakeholders in acute care and home care organizations across Canada.

**Results:**

Decision-makers (n = 22) and patients/families (n = 24) from five provinces participated in the Delphi. Following Round 1, 45 perceived value statements were identified. In Round 2, consensus was received on 33/45 (73.3%) by decision-makers, and 30/45 (66.7%) by patients/families. In Round 3, additional value statements reached consensus in the decision-makers’ survey (3) and in the patients/families’ survey (2). A total of 30 high priority value statements achieved consensus derived from both the decision-makers’ and patients/families’ perspectives.

**Conclusion:**

This study was an important first step in identifying key consensus-based priority value statements for monitoring care transitions from the perspective of both decision-makers and patients/families. Future research is needed to test their usability and to determine whether these value statements are actually suggestive of safe person-and family-centered care transition interventions from hospital to home.

## Background

Person- and-family-centered care (PFCC) allows the planning, delivery, and evaluation of health care to be grounded in mutually beneficial partnerships among health care providers, patients, and families[1]. Providers who believe in PFCC will typically develop relationships, communicate, collaborate, share information and engage with patients and their families with regards to health care[2]. Patient and family engagement is fundamental to both a PFCC approach to health delivery as well as to improving overall patient safety in our healthcare system[2–4]. This engagement process is defined as “patients, families, their representatives, and health professionals working in active partnership at various levels across the healthcare system—direct care, organizational design and governance, and policy making—to improve health and health care”[3], p. 224. It may encompass the complete spectrum of engagement, from understanding patients’ experiences and perspectives with the health system to fully involving patients and families in improving the health of their communities[3,5]. Research shows that patients who are more involved in the decision-making process related to their care are better able to manage complex chronic conditions,[6–8] have reduced anxiety and stress[9] and have shorter lengths of stay in hospital[10]. There is growing consensus that engaging patients and families can improve the quality of their care, particularly when transitioning from hospital to home[11].

PFCC practices during transitions in care are defined as: “a set of actions designed to ensure the coordination and continuation of health care as patients transfer between different locations or between levels of care within the same clinical setting”[12], p. 533. The challenges associated with care transitions are complex, and, thus require a multifaceted approach[13]. Existing care transition models such as the Care Transitions Interventions (CTI)[14] and the Transitional Care Model (TCM)[15] can provide insight into interventions that can best support patients during care transitions. Despite, the many studies that have trialed multifaceted care transition interventions to improve the patient experience and to reduce readmissions to hospital[16–20], the results have been inconsistent[21–25].

Thus, care transitions, particularly from hospital to home, continue to be poorly managed and pose a high risk for harm[26–29]. A recent systematic review of transitional care interventions (n = 12) for older people with chronic illnesses has shown the need for more involvement from patients and from their informal caregivers [30]. There is a need for additional research to focus on engaging patients and their families in identifying aspects of care transitions that are most important to them and that are linked to safe person-and family-centered. In this study, we used value statements as a way to obtain narratives in lay terms about how well care, treatment and support was organized to meet the needs and the preferences of patients and families[31]. Value statements were chosen over the more usual performance measures because they were easier for lay participants to understand[32]. The purpose of this study was to identify the value statements that are perceived by health care decision-makers, patients and families to best signify safe person- and family-centered care during transitions from hospital to home.

## Methods

We conducted a three-round, web-based Delphi technique[33] to obtain consensus on potential value statements that best reflect safe person- and family-centered care transitions. We obtained ethics approval for our study from the University of Ottawa Research Ethics Board (uOttawa REB), and the Ottawa Health Science Network Research Ethics Board (OHSN REB).

### Sampling strategies and recruitment

Key stakeholders including health care decision-makers from organizations across Canada, and patients/families chosen from a pan-Canadian patient advisory group were invited to participate. Decision-makers included: quality and/or performance measurement (decision support) leads, medicine and/or surgery directors and managers, and physician and nursing leadership from hospitals, regional health authorities or home care agencies who were involved in the oversight of initiatives related to improving care transitions from hospital to home. Decision-makers were either previously known to the research team for their expertise, or were identified through an Internet-based search of their organization website. Patient and family representatives were recruited through the primary contact within each of the participating organizations. Eligible patient and family representatives were defined as those 18 years of age or older with experience as a patient or as a primary caregiver or who had previously experienced a care transition for an acute trauma or an illness.

Email invitations were sent using the modified Dillman approach[34]. To recruit decision-makers, the email invitations consisted of an initial standardized recruitment message with a link to the survey. The message consisted of an invitation for the decision-makers to complete the survey or to share it with other decision-makers within their organization, as appropriate. To recruit patient and family representatives, a separate email invitation was sent via the decision-makers to specifically invite their patient and family advisory council members to complete the survey. At two and four weeks intervals after the initial invitation email, reminder emails were sent.

### Data collection

In **Round 1**, decision-makers were asked to participate in a web-based survey to identify the interventions and related measures in place in their organization, to monitor transitions from hospital to home. The specific questions are listed in Table 1.

**Table 1:** Delphi Round 1 survey questions.

**1**	What are the current practices you use to ensure overall quality and safety during care transitions from hospital to home?
**2**	For each of the current practices identified, who is your target patient population?
**3**	For each of the current care transition practices, what measure do you use to monitor the practice?
**4**	If you do not monitor the practice, how would you ideally monitor it (if you could)?

These results were gathered to develop the Round 2 survey. The research team also evaluated best practices based on previously published literature to identify other potential value statements.

In **Round 2**, all participants (decision-makers and patients/family members) were asked to rate on a scale of 1 to 5, each of the value statements according to their importance to overall quality and safety during care transitions from hospital to home. The scale corresponded to: (1) Not important, (2) Slightly important, (3) Moderately important, (4) Important, and (5) Very Important. Participants were also asked to suggest other value statements that they felt were missing from the list provided.

In **Round 3**, all participants (decision-makers and patients/family members) were asked to reconsider their answer regarding the value statements for which no consensus had been reached regarding their importance. Beside each statement, we provided the participants with the group response (decision-makers vs. patient/family members), and their own individual response to the value statement. We also requested that participants comment on their answers.

### Data analysis

The lead author (CB) led the data analyses. In **Round 1**, a trained research assistant and CB independently grouped the responses together in semantically similar themes, and removed any duplicate statements. This was done by grouping similar response statements, and then theming them. To identify any missing themes, the research assistant reviewed the themes against the existing relevant literature found in our recent systematic review on the effectiveness of PFCC transition interventions from hospital to home[35]. Missing themes were then added to the primary data collected during Round 1. From the common themes identified, the research assistant and CB developed value statements which were reviewed by the research team. This created a list of value statements for Round 2. In **Round 2** and **Round 3**, data were reported using descriptive statistics. The consensus scores looked at the number of respondents within a group (decision-makers or patient/family members) that rated the importance of a specific value statement at a value of 4.0 or higher. The consensus score was thus defined as follows: *Consensus = # of respondents scoring a statement above 4*.*0/total number of respondents*. For the purpose of this study, consensus on each item was pre-defined to be at least 90% agreement among survey participants. The means for each of the value statements were calculated and used to rank the statements in order from the most important to the least important. The mean of the value statement was simply the numerical mean calculated as follows: *Mean = Sum of importance scores / # of respondents*. The additional themes suggested by the participants in **Round 2** were also developed as value statements by the research team.

## Results

Twenty-two decision-makers (n = 22) and twenty-four patients/families (n = 24) from five Canadian provinces participated in the Delphi survey. The demographic characteristics of the participants for the three Delphi rounds can be found in Table 2.

**Table 2:** Participant characteristics.

	Round 1	Round 2*	Round 3**
**DECISION-MAKERS**	**8**	**14**	**4**
Administrators	8	12	4
Nursing leaders	0	0	0
Physician leaders	0	2	0
**Organization type**			
Hospital	0	4	0
Home care	5	5	3
Regional Health Authority	3	5	1
**Province**			
Alberta	1	2	0
Ontario	5	8	3
Manitoba	1	0	0
New Brunswick	1	0	0
British Columbia	0	2	0
Newfoundland	0	1	1
Quebec	0	1	0
**PATIENTS/FAMILIES**	**n/a**	**25**	**12**
Patients	n/a	16	7
Families/caregivers	n/a	9	5
**Province**			
Alberta	n/a	1	1
Ontario	n/a	8	5
Northwest Territories	n/a	1	0
New Brunswick	n/a	11	4
British Columbia	n/a	1	0
Nova Scotia	n/a	3	2

*All participants who were invited to Round 1 were invited again to participate in Round 2.

**Only participants who responded to Round 2 were invited to participate in Round 3.

### Round 1

Following the first round, a total of 45 value statements were suggested by participants. Duplicates were removed, providing a list of 36 possible value statements. With the addition of 9 value statements from the literature, a total of 45 value statements were available for Round 2. The complete list of value statements for decision-makers and for patients/families can be found in the [S1 Supporting Information].

### Round 2

We identified a high level of consensus (≥90%) by decision-makers for 33/45 (73.3%) of the value statements. The consensus score looked at the number of respondents within a group that rated the importance of a specific value statement at a value of 4.0 or higher. We also identified high levels of consensus (≥90%) by patients/families for 30/45 (66.7%) of the value statements. The value statements that received high level of consensus and the new value statements suggested by participants in Round 2 are available in the S1 Supporting Information.

### Round 3

In Round 3, the value statements that did not receive consensus (those statements that did not reach 90% consensus or higher) for decision-makers (n = 12) and for patients/families (n = 15) were surveyed again. In this round, we also asked the participants to rate the additional value statements suggested by the participants in Round 2. In Round 3, a total of 4 decision-makers and 12 patients/families participated and reconsidered their previous response in the context of the group response to each benchmark.

The consensus results of the Round 3 survey are summarized in Table 3 for decision-makers and Table 4 for patients/families. In the decision-makers’ Round 3, three additional value statements reached consensus. In the patient/family Round 3, two additional value statements reached consensus.

**Table 3:** Decision-makers’ Round 3 value statements.

Value statements	Round 2 Decision-makers’ Consensus level	Round 3 Decision-makers’ Consensus level (n = 4)	Comment Modified in Round 3 (i.e. unchanged, etc)
Patients report their family doctor received a summary of their hospital stay including a list of all medications and follow-up appointments	86%	100%	Consensus reached
Patients’ health conditions impact their usual activities	86%	93%	Consensus reached
Patients receive a visit from the home care nurse 24 to 48 hours after going home (if applicable)	86%	93%	Consensus reached
Patients are assessed for their need to be re-hospitalized	86%	86%	Unchanged
Patients’ family doctor and care providers or clinicians in the community receive the contact information of their hospital doctor	86%	86%	Unchanged
Patients have a clear understanding about all of their prescribed medications before leaving the hospital	86%	86%	Unchanged
Patients know what symptoms to watch for after going home and can name them when they receive the call from the hospital	86%	86%	Unchanged
Patients missed work or other regular activities as a result of their health	86%	86%	Unchanged
Patients experience fatigue that impacts on their usual activities	86%	86%	Unchanged
Patients satisfaction with their life including physical health, family, education, employment, wealth, religious beliefs, finance, and the environment is assessed	79%	79%	Unchanged
Patients who receive a call from the hospital to see how they were doing after going home	71%	71%	Unchanged
Patients able to describe their emotions, how they feel and think, and general interests in self or others	71%	79%	

**Table 4:** Patients/families Round 3 value statements.

Value statements	Round 2 Patient /family Consensus level	Round 3 Patient /family Consensus level (n = 12)	Comment Modified in Round 3 (i.e. unchanged, etc)
You have to be re-admitted to the hospital because your condition got worse	88%	92%	Consensus reached
Your family doctor, and other care providers or clinicians in the community receive the contact information of your hospital doctor	88%	92%	Consensus reached
You can clearly describe what you have been doing to self-manage your condition, medications (if any) and if any symptoms appeared	88%	88%	Unchanged
Your health condition impacts your usual activities	83%	88%	
Your pharmacist received a list of all your medications at discharge from hospital	83%	88%	
You knew what symptoms to watch for after going home and can name them when you receive the call from the hospital	83%	88%	
You describe some symptoms or complications that may or may not be linked with medication or other treatment you received while at home or community facility	83%	88%	
You went to the emergency department because your condition got worse	79%	88%	
Your ability to function impacts on your usual activities	79%	79%	Unchanged
You are told what your risk/chance is for being re-hospitalized	75%	75%	Unchanged
You can describe your emotions, how you feel and think, and general interests in self or others	68%	72%	
Your satisfaction with your life including physical health, family, education, employment, wealth, religious beliefs, finance and the environment is assessed	63%	67%	
You experience fatigue that impacts on your usual activities	63%	58%	
You receive a call from the hospital to see how you were doing after going home	58%	58%	Unchanged
You missed work or other regular activities as a result of your health	58%	54%	

The value statements that received high level of consensus in Round 3 are available in the S1 Supporting Information.

Decision-makers and the patients/families’ means and consensus levels were then compared to identify which value statements were deemed important (mean>4.0) and obtained consensus (>90%) in each of the groups. The value statements that were important to both group include the following:

1. Patients’ preferences about their healthcare needs is considered prior to leaving the hospital
2. Patients’ medications are checked and confirmed when they are admitted to the hospital)
3. Patients’ family doctors (or primary health care team) are notified by the hospital care team that they are in the hospital
4. Patients’ ability to get and understand the information about their health and services they need for making appropriate decisions regarding health care services is assessed by the hospital care team
5. Patients’ plan of care includes information about what will happen or what they need if they are discharged to go home or to another community facility
6. Patients are involved in discussion about their preferences about end-of-life care (as applicable)
7. Patients feel they have the supports in place to leave the hospital
8. Patients who report that during this hospital stay, the doctors/nurses or other hospital staff talked with them about whether they would have the help they needed when they left the hospital
9. Patients whose readiness to go home was assessed by their hospital care team
10. Patients receive written (or electronic) information about the care they received in hospital and how to care for themselves before going home or to a community facility
11. Patients report that their family doctor received a summary of their hospital stay including a list of all their medications and follow-up appointments
12. Patients are given a date and time for a follow-up appointment with a specialist of their family doctor (if recommended by their hospital care team)
13. Patients are made aware of the tests that need to be done after going home (if necessary)
14. Patients arrangements are made during their hospital stay so they are provided with the necessary services and equipment before leaving the hospital
15. Patients’ medications are checked and confirmed as appropriate and accurate before leaving the hospital
16. Patients receive adequate/ appropriate verbal and written information from hospital staff about what to do (including who to call) if symptoms to watch for appear after leaving the hospital
17. Patients receive the name and contact information of the person to contact if they needed help after going home
18. Patients understands their care before leaving the hospital
19. Patients had a nurse or other home care service come see them at home within the time frame that they were told (if required)
20. Patients have the help they need after going home
21. Patients experience pain that impacts on their usual activities
22. Patients able to describe an alarming reaction they had to the medication or other treatment while at home or community facility (if needed)
23. Patients assessed for the risk of falls after going home
24. Patients re-admitted to the hospital because their condition got worse
25. Patients’ family doctors are informed and up-to-date about the care provided by patients’ specialist(s)
26. Patients are contacted about the home care services that they will receive after going home (if required)
27. Patients’ home care nurses assessed their health needs (if applicable)
28. Patients’ medications are checked and confirmed in their home or community facility
29. Patients report their family doctor received a summary of their hospital stay including a list of all medications and follow-up appointments
30. Patients receive a visit from the home care nurse 24 to 48 hours after going home (if applicable)

## Discussion

This study resulted in a consensus-based list of value statements associated with safe person- and family-centered care transitions from hospital to home, derived from both the decision-makers and patients/families’ perspectives. The high priority value statements which achieved consensus across both patients/families and decision-makers’ groups (n = 30) represent key elements of care transitions that could enable patients as well as decision-makers to determine if their transition was successful or not. These statements are foundational to building a learning system that can grow and improve with each patient care episode. The value statements that reached consensus (>90%) and were important (mean>4.0) in both groups spanned the full transition experience from hospital admission to post-discharge follow up.

We acknowledge the relatively limited attention span and ‘improvement fatigue’ in health care delivery. Presenting a list of 30 items for care improvement will likely be met with considerable push back. One strategy to counter this might be to examine the current list through the lens of what metrics/performance indicators may already address some of the recommended/high concordance approaches. This would help with prioritization of the recommendations.

Additionally, several value statements that were identified as a high priority for decision-makers were not priorities for patients/families, specifically the patient-reported outcome measures (PROMs) including: health status, activities of daily living/social activities, missed work, quality of life, symptoms (fatigue), and distress (anxiety or depression). This appears to be consistent with the work of others[36] where patients have shown less interest in measures that are dependent on their own subjective factors rather than system/provider factors. Furthermore, the ‘post-discharge phone call’ value statement did not reach consensus and it was not identified as being important by both groups. With the increased focus on patients and families’ involvement as active partners on the healthcare team, it is important that they are part of the priority setting for care transitions as their involvement will influence the improvement efforts[37].

To our knowledge, there has been no previous research that has focused on identifying value statements to monitor safe person- and family-centered care transition interventions. Our study was also focused on engaging patients and their families, and specifically used instruments- value statements- which were more accessible to people that were not in the health care field, as this engagement is an integral aspect of person- and family-centered care. This allowed us to gain rich insights into the perspectives of both the decision-makers and the patients and their families. Moreover, these experts were gathered from both home care organizations and acute care settings across five provinces across the country. Another strength of this study was that it was done in multiple rounds over a lengthy period of time. The passage of time and potentially changing levels of health can alter an individual’s perception of which statements are most important. This allowed patients/families time to truly reflect on the most valuable statements and change their responses.

The Delphi technique proved helpful in systematically identifying and gaining consensus, on a core set of appropriate priorties from patients/families and decision makers where no consensus had previously existed. Careful consideration was necessary in relation to understanding the Delphi process, identification of ‘experts’, questionnaire design, agreement on an appropriate level of consensus and the number of rounds to conduct.

The 30 value statements on which consensus was gained will enable patients/families and decision makers to identify gaps in their practice against their peers, encourage improvement in care transitions and establish ‘standards’ of what types of care are feasible. The value statements may also be used by health care providers to monitor, evaluate, and improve the quality of care provided during care transitions. It is important that these priorities are linked with other research initiatives aimed at addressing the quality of care transitions as a whole. Although this study is an important step towards routinely measuring the quality of care provided to patients during care transitions, it is important that it is incorporated into a process of continuous quality improvement.

Because the respondents were based across Canada, it was not possible to meet face to face in a consensus conference or to take part in nominal groups. Therefore, the web-based Delphi approach was selected as the most appropriate and the most relevant research approach. Although we sought representation across Canada, not all experts responded to our invitations to participate in the survey. Direct care providers (i.e, nurses, physicians, or social workers) who are involved in discharge and care transition planning were not involved in the study. Furthermore, it is possible that patients/families who participated had greater interest in their health and in promoting change due to their involvement as patient and family advisors. Thus, their rating of the level of importance of each value statement could be higher. The patients/families could also have been a healthcare professional in the past.

The web-based Delphi worked well in this study. The reason for this may be because most of the experts identified in the Delphi sample had easy access to e-mail and they use it as the main form of communication. The advantages of the web-based Delphi are obvious; not only is it an environmentally friendly way to carry out research, it also lead to more rapid responses from participants and it sped up the analysis as the electronic responses could be directly uploaded into SPSS. In addition, reminder e-mails can be sent out automatically, and there is no cost in terms of postage or printing. It is also possible that an electronic questionnaire where the busy respondent sees one page at a time is perceived as being easier to commence than a full printed questionnaire. The disadvantages include the possibility that not all Delphi experts would have an e-mail account—although this is getting less common. Furthermore, as with all questionnaires it is possible that busy people will complete the web-based Delphi in a casual fashion or may decide not to participate. For some managers who may be potential Delphi experts, it is often the case that their secretaries or personal assistants have access to their e-mail accounts and this may threaten response anonymity. It is important that all e-mails are labelled as strictly private and confidential. Finally, the sensitivity of computer firewalls in some organisations may block web-based Delphi questionnaires or direct them into a junk folder.

Next steps in this research will be to work alongside other organizations to help inform the ongoing development of value statements, interventions and standards that are meaningful and reflect the perspectives of both decision-makers and patients and their families. The consensus-based list of value statements found in this study can also inform the development of safe person- and family-centered care transition interventions as many health care organizations are seeking to develop and prioritize improved care transitions as part of their quality rubric. Further research should also focus on the barriers that different organizations and systems may face in operationalizing these value statements.

## Conclusion

The present study was an important first step in identifying key consensus-based priority value statements for monitoring care transitions from hospital to home from the perspective of both the decision-makers and the patients/families. Further research is needed to test their usability and to determine whether these value statements are actually suggestive of safe and effective person- and family-centered care transition interventions from hospital to home.

## Supporting information

S1 Supporting Information List of value statements for decision-makers and for patients/families. (DOCX)
