# Supplementary material for: Safe and effective person- and family-centered care practices during transitions from hospital to home—A web-based Delphi technique
Source: PLoS One. 2019 Jan 22;14(1):e0211024. doi: 10.1371/journal.pone.0211024 (PMC6342305; doi:10.1371/journal.pone.0211024)
Supplement: S1 Supporting Information — (DOCX) [file pone.0211024.s001.docx]

**S1 Supporting Information**

**List of value statements for decision-makers and for patients/families**

**Legend:**

*New value statements suggested by participants in Round 2

R2 = value statement with high level of consensus (>90%) in Round 2

R3 = value statement with high level of consensus (>90%) in Round 3

| **Concepts** | **Potential indicators** | **Value statements**  **(Decision-Makers)** | **Value statements (Patients/families)** |
| --- | --- | --- | --- |
| **ON ADMISSION TO HOSPITAL** |  |  |  |
| **Readmission risk factor screen** | Hospital readmission risk | Patients are assessed for their need to be re-hospitalized | You are told what your risk/chance is for being re-hospitalized |
| **Reason for hospitalization** |  | *Patients are aware of the current reason for hospitalization and understand the relationship with their other health conditions **(R3)** | *You are aware of the current reason for your hospitalization and you understand the relationship with your other health conditions **(R3)** |
| **Discharge planning** |  | *Discharge planning is started as soon as it is known a patient will be admitted | *Your discharge plan is started as soon as it is known you will be admitted |
| **Discharge needs analysis** | Admissions where patients are included in identifying post-discharge needs | Patients’ preferences about their healthcare needs are considered prior to leaving the hospital **(R2)** | Your preferences about your healthcare needs are considered before you leave the hospital **(R2)** |
| **Medication reconciliation on admission** | Medication reconciliation is completed on patient’s admission to the hospital | Patients’ medications are checked and confirmed when they are admitted to the hospital **(R2)** | Your medications are checked and confirmed when you are admitted to the hospital **(R2)** |
| **Communication with primary care provider** | Patients’ notifications of hospitalization are sent to primary care providers at the beginning of the hospital visit (documented) | Patients’ family doctors (or primary health care team) are notified by the hospital care team that they are in the hospital **(R2)** | Your family doctor (or primary health care team) is notified by the hospital care team that you are in the hospital **(R2)** |
| **Health literacy** | Health literacy score | Patients’ ability to get and understand the information about their health and services they need for making appropriate decisions regarding health care services is assessed by the hospital care team **(R2)** | Your ability to get and understand the information about your health and services you need for making appropriate decisions regarding health care services is assessed by the hospital care team **(R2)** |
| **DURING HOSPITALIZATION** |  |  |  |
| **Individualized care and transition or discharge care plans** | Patients with a documented transition or discharge plan incorporated into their plan of care | Patients’ plan of care includes information about what will happen or what they need if they are discharged to go home or to another community facility **(R2)** | Your plan of care includes information about what will happen or what you need if you are discharged to go home or to another community facility **(R2)** |
| **Patient engagement in plan of care** |  | *Patients are involved in developing their plan of care to the extent that they desire **(R3)** | *You are involved in developing your plan of care to the extent that you desire **(R3)** |
| **Hospital end-of-life discussions (if applicable)** | Patients with whom in hospital end-of-life discussions took place | Patients are involved in discussion about their preferences about end-of-life care (as applicable) **(R2)** | You are involved in discussion about your preferences about end-of-life care (as applicable) **(R2)** |
| **Transition or Discharge readiness** | Patients with supports and interventions in place to ensure their readiness for transition or discharge (includes preferences and needs)          Patients with discharge readiness assessed including seeking and documenting patient readiness | Patients feel they have the supports in place to leave the hospital **(R2)**    Patients who report that during this hospital stay, the doctors/nurses or other hospital staff talked with them about whether they would have the help they needed when they left the hospital **(R2)**    Patients whose readiness to go home was assessed by their hospital care team **(R2)** | You feel you have the supports in place to leave the hospital **(R2)**    During this hospital stay, doctors/nurses or other hospital staff talked with you about whether you would have the support/help you needed when you left the hospital. **(R2)**        Your readiness to go home is assessed by your hospital care team **(R2)** |
|  |  | *Healthcare providers have clear written methods of communicating with each other and with patients the information required for discharge planning **(R3)** | *Healthcare providers have clear written methods of communicating with each other and with you the information required for discharge planning **(R3)** |
| **AT DISCHARGE FROM HOSPITAL** |  |  |  |
| **Discharge information** | Patients receiving a written or electronic discharge summary for transition to other setting or healthcare providers | Patients receive written (or electronic) information about the care they received in hospital and how to care for themselves before going home or to a community facility **(R2)** | You received written (or electronic) information about the care you received in hospital and how to care for yourself before going home or to a community facility **(R2)** |
| **Timely handover communication to primary care provider** | Time between discharge and transmission of summary (all medications and follow-up appointments) to primary healthcare provider | Patients report their family doctor received a summary of their hospital stay including a list of all their medications and follow-up appointments **(R3)** | Your family doctor received a summary of your hospital stay including a list of all your medications and follow-up appointments **(R2)** |
| **Communication of medication list to pharmacist** | Patients with a discharge medication list sent to pharmacy | Patients’ pharmacists receive a list of all their medications at discharge from hospital **(R2)** | Your pharmacist received a list of all your medications at discharge from hospital |
| **Post-hospital care follow-up appointment pre-booked (as required)** | Discharged patients who had follow-up visits scheduled before being discharged in accordance with their level of assessed risk | Patients are given a date and time for a follow-up appointment with a specialist of their family doctor (if recommended by their hospital care team) **(R2)** | You were given a date and time for a follow-up appointment with a specialist or your family doctor (if recommended by your hospital care team) **(R2)** |
| **Plan for the follow-up of results from tests or labs that are pending at discharge** | Patients after-hospital care plans include pending tests and follow-up | Patients are made aware of the tests that need to be done after going home (if necessary) **(R2)** | You were made aware of the tests that need to be done after going home (if necessary) **(R2)** |
| **Organize post-discharge outpatient services and medical equipment** | Patients’ discharge summaries document arrangements made for necessary services and equipment | Patients arrangements are made during their hospital stay so they are provided with the necessary services and equipment before leaving the hospital **(R2)** | Arrangements are made during your hospital stay so you are provided with the necessary services and equipment before leaving the hospital **(R2)** |
|  |  | *The patient's family members are involved in the plan of care and have been educated and are prepared for the patient's current and future needs **(R3)** | *Your family members are involved in the plan of care and have been educated and are prepared for your current and future needs **(R3)** |
| **Medication reconciliation at discharge** | Patients medication reconciliation is completed at discharge | Patients’ medications are checked and confirmed as appropriate and accurate before leaving the hospital **(R2)** | Your medications are checked and confirmed as appropriate and accurate before leaving the hospital **(R2)** |
| **Provide verbal and written information and confirm level of understanding with patient regarding any potential symptoms to watch for after discharge** | Patients were provided verbal and written information on what to do and who to call if and when symptoms to watch out for appeared after discharge | Patients receive adequate/ appropriate verbal and written information from hospital staff about what to do (including who to call) if symptoms to watch for appear after leaving the hospital **(R2)** | You receive adequate/ appropriate verbal and written information from hospital staff about what to do (including who to call) if symptoms to watch for appear after leaving the hospital **(R2)** |
| **Who to contact: Provide information and confirm level of understanding** | Patients receive the contact information of whom to seek help after discharge | Patients receive the name and contact information of the person to contact if they needed help after going home **(R2)** | You receive the name and contact information of the person to contact if you needed help after going home **(R2)** |
| **Communication between hospital clinicians' and receiving clinicians upon discharge** | Patients whose hospital provider contact information was provided to receiving clinicians upon discharge | Patients’ family doctor and care providers or clinicians in the community receive the contact information of their hospital doctor | Your family doctor, and other care providers or clinicians in the community receive the contact information of your hospital doctor **(R3)** |
| **Self-care or self-management plan: Provide information and confirm level of understanding** | Patients understand their self-care or self-management plans | Patients understands their care before leaving the hospital **(R2)** | You have a good understanding of your care before leaving the hospital **(R2)** |
|  |  | *Patients have a good understanding of the things that their healthcare providers will do to manage their health during their stay in the hospital and after returning to the community | *You have a good understanding of the things that your healthcare providers will do to manage your health during your stay in the hospital and after you return to the community **(R3)** |
| **Understand medications** | Patients with a clear understanding about their medications | Patients understand their medications before leaving the hospital | You have an understanding about your medications before leaving the hospital **(R2)** |
| **HOME OR COMMUNITY FACILITY** |  |  |  |
| **Post-discharge phone call** | Patients with follow-up phone calls completed within 48 to 72 hours of discharge | Patients who receive a call from the hospital to see how they were doing after going home | You receive a call from the hospital to see how you were doing after going home |
| **Increased knowledge of symptoms to watch for as part of self-management** | Patients (or family member/caregiver) who report correctly the symptoms to watch for during the post-discharge phone call | Patients know what symptoms to watch for after going home and can name them when they receive the call from the hospital | You knew what symptoms to watch for after going home and can name them when you receive the call from the hospital |
| **Increased knowledge or ability to self-manage** | patients who clearly describe how they have been self-managing including when symptoms appeared | Patients can clearly describe what they have been doing to self-manage their conditions, medications (if any) and if any symptoms appeared **(R2)** | You can clearly describe what you have been doing to self-manage your condition, medications (if any) and if any symptoms appeared |
| **Appropriate services (i.e. home care services)** | Time from discharge to home care nursing visit for high-risk patients | Patients had a nurse or other home care service come see them at home within the time frame that they were told (if required) **(R2)** | You had a nurse or other home care service come see you at home within the time frame that you were told (if required) **(R2)** |
| **Access to appropriate help (i.e. supports or care providers/caregivers) at home or community facility** | Patients with appropriate supports in place after discharge | Patients have the help they need after going home **(R2)** | You have the help you need after going home **(R2)** |
| **Missed work** | Patients who had to miss work due to their physical health | Patients missed work or other regular activities as a result of their health | You missed work or other regular activities as a result of your health |
| **Quality of life** | Quality of life score | Patients satisfaction with their life including physical health, family, education, employment, wealth, religious beliefs, finance, and the environment is assessed | Your satisfaction with your life including physical health, family, education, employment, wealth, religious beliefs, finance and the environment is assessed |
| **Health status** | Health status score | Patients’ health conditions impact their usual activities **(R3)** | Your health condition impacts your usual activities |
| **Activities of daily living/social activities** | Functional status score | Patients’ ability to function impacts on their activities of daily living **(R2)** | Your ability to function impacts on your usual activities |
| **Symptoms (Pain or fatigue)** | Patients experience with symptoms (pain, fatigue) that impact on their activities of daily living | Patients experience fatigue that impacts on their usual activities    Patients experience pain that impacts on their usual activities **(R2)** | You experience fatigue that impacts on your usual activities    You experience pain that impacts your usual activities **(R2)** |
| **Distress (anxiety or depression)** | Depression Index Score | Patients are able to describe their emotions, how they feel and think, and general interests in self or others | You can describe your emotions, how you feel and think, and general interests in self or others |
| **Adverse events** | Harm or near harm incident resulting from care, treatment or medication | Patients able to describe an alarming reaction they had to the medication or other treatment while at home or community facility (if needed) **(R2)** | You are able to describe an alarming reaction you had to the medication or other treatment while at home or community facility (if needed) **(R2)** |
| **Post discharge complications** | Patients with complications or symptoms of known or unknown cause after discharge | Patients describe some symptoms or complications that may or may not be linked with medication or other treatment they received while at home or community facility **(R2)** | You describe some symptoms or complications that may or may not be linked with medication or other treatment you received while at home or community facility |
| **Fall risk assessment** | Patients screened for fall risk on initial home care visit    Patients deemed high risk if they received intervention | Patients assessed for the risk of falls after going home **(R2)**    Patients are provided personalized approaches to prevent falling (as required) **(R2)** | You were assessed for your risk of falls/falling after going home **(R2)**    You are provided personalized approaches to prevent falling (as required) **(R2)** |
| **30-day all cause readmissions** | Discharges with readmission for any cause within 30 days | Patients re-admitted to the hospital because their condition got worse **(R2)** | You have to be re-admitted to the hospital because your condition got worse **(R3)** |
| **Emergency Department visits within 30 days of hospital discharge** | Patients visiting the emergency department within 30 days of hospital discharge | Patients went to the emergency department because their condition got worse **(R2)** | You went to the emergency department because your condition got worse |
| **Primary care provider informed and up-to-date about the care from specialists** | Patients reported how often their primary care provider seemed informed and up-to-date about the care from specialists | Patients’ family doctors are informed and up-to-date about the care provided by patients’ specialist(s) **(R2)** | Your family doctor is informed and up-to-date about the care you received from your specialist(s) **(R2)** |
| **Connection to home care services** | Patients connected with home care services        Patients receive nursing visit 24-48 hours post-discharge (as required) | Patients are contacted about the home care services that they will receive after going home (if required) **(R2)**    Patients receive a visit from the home care nurse 24 to 48 hours after going home (if applicable) **(R3)** | You are contacted about the home care services that you will receive after going home (if required) **(R2)**    You receive a visit from the home care nurse 24 to 48 hours after going home (if applicable) **(R2)** |
| **Standardized clinical assessment by home care provider** | Patients referral to homecare services based on completed standardized clinical assessment | Patients’ home care nurses assessed their health needs (if applicable) **(R2)** | Your home care nurse assessed your health needs (if applicable) **(R2)** |
| **Medication reconciliation in home setting or community facility by home care services** | Patients’ medication reconciliation completed in home setting or community facility | Patients’ medications are checked and confirmed in their home or community facility **(R2)** | Your medications are checked and confirmed in your home or community facility **(R2)** |
| **Caregiver safety** |  | *Patient's compatibility with their home environment is assessed (as required) and suggestions are made to accommodate or reorganize the environment so the patient can function at home despite physical/health limitations **(R3)** | *Your compatibility with your home environment is assessed (as required) and suggestions are made to accommodate or reorganize the environment so you can function at home despite physical/health limitations **(R3)** |
|  |  | *Clear verbal or written communication between care providers allows for consistency of the information provided to the patient regarding their condition and healthcare needs **(R3)** | *Clear verbal or written communication between care providers allows for consistency of the information provided to you regarding your condition and healthcare needs **(R3)** |
| **Experience with home care team** |  | *Patients are provided opportunities to express satisfaction, or areas in need of improvement, regarding their experiences throughout their care **(R3)** | *You are provided opportunities to express satisfaction, or areas in need of improvement, regarding your experiences throughout your care **(R3)** |
